# Investigating the impact of 1 hour of daily outdoor access on the gait and hoof health of nonclinically lame cows housed in a movement-restricted environment

**DOI:** 10.3168/jdsc.2023-0498

**Published:** 2024-03-08

**Authors:** A. Nejati, E. Shepley, G.M. Dallago, E. Vasseur

**Affiliations:** 1 Department of Animal Science, McGill University, Sainte-Anne-de-Bellevue, Quebec, Canada, H9X 3V9; 2 Department of Veterinary Population Medicine, University of Minnesota, St. Paul, MN 55108; 3 Department of Animal Science, University of Manitoba, Winnipeg, Manitoba, R3T 2N2, Canada

## Abstract

Tiestalls, known for restrictive housing, can reduce cows' locomotor skills over time. While outdoor access benefits clinically lame cows, its effect on nonclinically lame cows is less known. This study evaluated 1 h daily outdoor access on gait and hoof health of nonclinically lame Holstein cows in tiestalls. Thirty cows, blocked by parity and DIM, were assigned to exercise (1 h outdoor access 5 d/wk for 5 wk) or nonexercise groups. Visual scoring assessed 6 gait attributes and overall gait (on scales of 0–5 and 1–5, respectively) at pre-trial, post-trial, and 8-wk follow-up stages. A total of 15 cows (9 exercise, 6 nonexercise) underwent visual gait scoring, with logistical challenges and exclusion criteria leading to this selection. Hoof health for all 30 cows was evaluated during pre-trial and follow-up hoof trims, documenting claw lesions. Hoof thermography captured dorsal coronary band views in wk 1 and 5. No significant gait score changes were observed, but exercise cows showed a 1-point improvement in overall gait score and 3 gait attributes after 5 wk of outdoor access, which persisted at follow-up. Sole hemorrhages were the only claw lesions observed, and their prevalence and severity remained consistent between pre-trial and follow-up for both groups. Thermography showed consistent coronary band temperature metrics across groups and over time. In conclusion, brief outdoor sessions resulted in noticeable, albeit not statistically significant, improvements in the gait of nonclinically lame cows in restrictive housing settings without adverse hoof health effects. Further studies should evaluate different outdoor access benefits and use precise gait and hoof health analysis technologies for a more accurate detection of subtle changes.

## Highlights

• Outdoor access may enhance gait, suggesting a 1-score boost in confined cows.
• Hoof lesion examinations showed outdoor access does not lead to hoof health risks.
• Using thermography to monitor hoof temperature indicated that outdoor sessions preserve hoof health.

## Graphical Abstract

**Figure:**
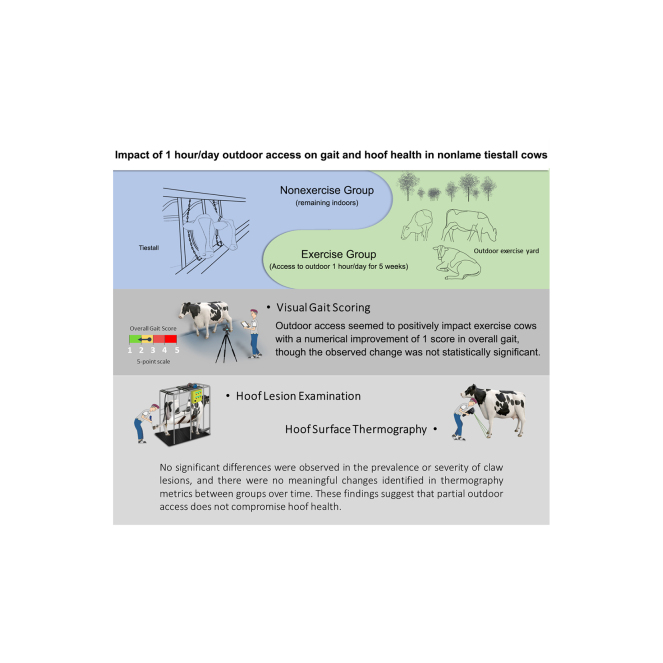
**Summary** In this study, the impact of 1 hour/day of outdoor access over 5 weeks on the gait and hoof health of nonclinically lame lactating Holstein cows in tiestalls was investigated. Of the 30 cows, half were granted outdoor access (exercise), whereas the remainder stayed indoors (nonexercise). Assessments included visual gait scoring, claw lesion assessment, and hoof thermography. Although changes in gait scores did not show significant group differences, the exercise group exhibited a numerical improvement of 1 score in overall gait on a 5-point scale by the end of the study. Claw lesion records, both in prevalence and severity, and hoof thermography metrics remained unchanged between groups throughout the study. In conclusion, short outdoor sessions seem to positively influence gait in movement-restricted cows without detrimental effects on hoof health.

Indoor housing systems for cows vary in confinement levels (Shepley et al., 2020). Tiestalls are the most restrictive, limiting voluntary movement and social behaviors (Popescu et al., 2013). This continuous level of restriction can reduce locomotor abilities over time, even without leading to clinical lameness (Shepley and Vasseur, 2021a). Providing additional space can improve cow health and welfare (Popescu et al., 2013; Shepley et al., 2020; Palacio et al., 2023). This additional space may take the form of pasture access, where cows can graze, or exercise yards, which provide enhanced opportunities for movement (Smid et al., 2020).

Little is known about how providing enhanced opportunities for movement affects the gait of dairy cows. The few studies available either center their focus on lame animals, highlighting the benefits of outdoor access for improving their condition, were conducted in freestalls, or were assessed for year-round or seasonal access to pasture (Hernandez-Mendo et al., 2007; Chapinal et al., 2013). As a result, there is limited understanding of how improvements in movement opportunities provided via short exposures to outdoor yards affect the gait of nonclinically lame, movement-restricted cows in tiestalls. The objective of this study was to investigate the impact of enhancing movement opportunity through the provision of 1 h of daily access to an outdoor exercise yard on the gait and hoof health of nonclinically lame lactating Holstein cows housed in tiestalls. We hypothesize that cows given daily outdoor access for 5 wk will improve their gait compared with those without access. We also anticipate no negative impact on hoof health, assessed by hoof lesion prevalence, severity, and surface temperatures.

This study was part of a larger study conducted at the Macdonald Campus Dairy Unit (McGill University, Ste-Anne-de-Bellevue, QC, Canada) evaluating the effect of outdoor access on cows' welfare and behavior. Ethical animal use was certified by the Animal Care Committee of McGill University and affiliated hospitals and research institutes (#2016–7794). Thirty tiestall-housed lactating Holstein cows, representative of typical herd DIM and parity, were enrolled. Cows were selected based on locomotor functionality, excluding those with ulcerative hoof lesions, clinical lameness (≥4 on a 5-point scale), or conformation issues. Cows were grouped into 5 blocks (n = 6/block) by parity (average 2.1, range 1–4) and stage of lactation (166.3 ± 11.9 DIM). Within each block, 3 cows were randomly assigned to the exercise treatment (1 h/d outdoor access, Monday–Friday, for 5 wk; n = 15) and 3 to the nonexercise treatment (remaining indoors; n = 15). Exercise cows were moved to 1 of 5 randomly assigned rectangular outdoor exercise paddocks, each measuring 117 m^2^ (9 m × 13 m), located 50 m from the barn. Conducted in November and December, the study faced temperatures ranging from −13°C to 10°C, with snow and frozen surfaces. Surfaces varied from soft grass and sand to uneven and hard terrain, with fresh sand applied for traction when needed. Blocks rotated weekly through all paddocks. No treatments or medications were required during the study. Three main measures (visual gait scoring, claw lesion assessment, and hoof thermography) were assessed during 3 data collection periods: (1) pre-trial, at the start of the study, (2) post-trial, at the end of the study, and (3) follow-up, 8 wk after the end of the experiment.

Due to logistical and time constraints, a subsample of 20 cows (4 cows/block) were randomly selected for gait analysis. Cows were led by halter to a designated motion analysis area, equipped with high-speed cameras and an 8-m test corridor covered with 1.9-cm-thick rubber mats (Ani-Mats). Each cow made at least 5 passages, ensuring a consistent walking pace without running or stopping for at least one passage. Cows that did not meet these criteria were excluded, resulting in 15 cows (9 exercise, 6 nonexercise) for gait analysis. For this study, only visual gait scoring data were used. Forty-five recorded videos (15 cows at 3 periods) from one side-view camera (perpendicular to the cows' walking direction) were randomized and scored by a trained observer blinded to treatment. Six gait attributes were scored: swinging out, back arch, tracking up, joint flexion, asymmetric gait, and reluctance to bear weight (Shepley and Vasseur, 2021a), on a 0-5 scale with 0.5 intervals. Overall gait was scored on a 1–5 scale with 0.5 intervals, where 1 represented the soundest gait and 5 indicated severe lameness (Flower and Weary, 2006). Gait was analyzed by comparing changes in scores from post-trial and pre-trial and from follow-up and pre-trial.

Clinical hoof assessment for all cows was conducted at pre-trial and follow-up. This timing aligns with the time-dependent progression of laminitis pathogenesis, where the effects of metabolic and mechanical stressors typically become visible on the hoof sole after an 8-wk period (Shearer et al., 2015). Claw lesions were classified according to the ICAR Claw Health Atlas (Egger-Danner et al., 2014) and recorded using a sheet indicating specific claw zones (adapted from Shearer et al., 2004). Lesion severity was scored on a 5-point scale (Nikkhah et al., 2005; Flower and Weary, 2006): 0 for no hemorrhages or discoloration, 1 for slight, 2 for moderate, 3 for severe hemorrhagic lesion, and 4 for sole ulcer (exposed corium).

Hoof thermography images (n = 480) were collected from the dorsal view of all 4 feet of all cows, with assessments conducted twice during wk 1 and 5 of the trial, before cows were moved outside. Thermal images were taken using a FLIR E4 upgraded to E8 firmware infrared thermal imaging camera (emissivity: 0.98; distance: 1 m; reflected temperature: 20°C) with cows standing in their stalls for at least 10 min before imaging. To reduce the impact of dirtiness on thermography analysis, only images of clean or slightly dirty feet (Schreiner and Ruegg, 2003) were included. After excluding 47 images, 433 images were retained for further analysis. Ambient temperature was collected by 4 Onset HOBO MX2300 Temperature/RH Data Loggers (Onset Computer Corporation, Bourne, MA). Thermal images underwent direct thermal analysis using Therma-CAM Researcher Professional 2.10 software (FLIR Systems Inc., Wilsonville, OR). Two regions of interest (**ROI**) were selected: the coronary band (**CB**) area and a control skin area above the CB. Four thermal variables were extracted from the 2 ROI: CB maximum temperature (**CB-Max**), CB mean temperature (**CB-Mean**), CB standard deviation (**CB-SD**), and temperature difference (**ΔT**) between CB-Max and the mean temperature of the skin control area. ΔT considered within-animal temperature differences rather than absolute values (Nikkhah et al., 2005; Alsaaod and Büscher, 2012). In addition, to further counter the influence of ambient temperature, which is a known factor affecting temperature measurements in infrared imaging (Alsaaod and Büscher, 2012), the thermal images were also subjected to another method, termed normalization. This included image processing and data extraction using Matlab (R2021a, MathWorks Inc., Natick, MA). Image processing involved cropping a square area with CB at the bottom third and skin above CB at the top two-thirds, conversion to grayscale, double precision, and pixel normalization to a 0–1 scale (Figure 1). Four statistical features (mean, SD, skewness, kurtosis) were extracted from each processed image (Al-Obaidy, 2016; Nejati, 2021).

**Figure 1:**
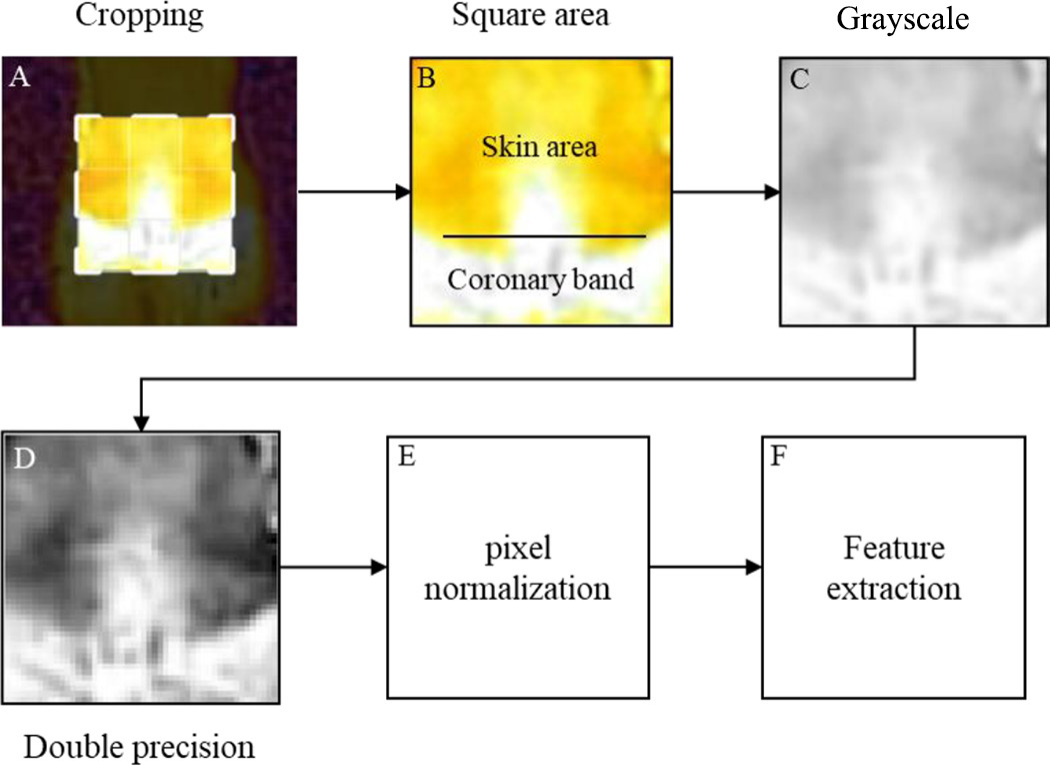
Normalization method applied to thermal images, showcasing its various pre-processing stages, including segmenting a square area (A) that includes the coronary band and the skin area (B), conversion to grayscale (C), transition to double precision (D), pixel normalization (E), and extraction of statistical features (F).

Statistical analysis was conducted using R (R Core Team, 2021). Claw lesions were treated as a binary variable and analyzed using a mixed effect logistic model with block (1 to 5), treatment group (nonexercise and exercise), period (pre-trial and follow-up), and the interaction between treatment and period as fixed effects and claw nested within cow as a random effect. Thermographic data were analyzed using a mixed effects model having block, limb position (fore and hind), treatment group, and week (1 to 5), as well as an interaction between treatment group and week, as fixed effects; limb (1 to 4) nested within cow as a random effect; and ambient temperature as a covariate. However, to analyze thermographic data through normalization, the ambient temperature was not included in the model. The overall gait score and 6 gait attributes were analyzed using a mixed effect model having block, treatment group, period difference (post-trial minus pre-trial and follow-up minus pre-trial), as well as an interaction between treatment group and period difference as fixed effects, and cow as a random effect.

Residual analysis was conducted for all models to evaluate the assumptions that the within-group errors were homoscedastic, independent, and followed a normal distribution and to determine that the random effects were normally distributed and independent. Heteroscedasticity was modeled by using a variance model with different variances for each level of a stratification variable. General, autoregressive of order 1, and compound symmetry correlation structures were also evaluated to model the possible time-dependencies among observations. Significance was declared at α < 0.05. Estimated effects were evaluated using marginal means (i.e., LSM) with Bonferroni *P*-value adjustment for multiple comparisons.

The average overall gait score of exercise cows numerically decreased from 2.78 ± 0.28 (SEM) at pre-trial to 1.78 ± 0.17 and 1.72 ± 0.17 at post-trial and follow-up, respectively, whereas the overall gait score of nonexercise cows changed from 2.92 ± 0.2 at pre-trial to 2.83 ± 0.21 and 2.5 ± 0.29 at post-trial and follow-up, respectively (Figure 2). The same numerical decrease occurred for 3 gait attributes: tracking up, asymmetric steps, and reluctance to bear weight (Figure 3). However, neither the overall gait score nor any of the 6 gait attributes differed significantly between treatment groups or periods (*P* > 0.05). It is worth noting that no cows in our study were obviously lame (gait score ≥4) at any point during the study period. Regarding lameness prevalence, at pre-trial a total of 8 cows (5 exercise and 3 nonexercise cows) were scored as moderately lame (gait score = 3 or 3.5). At post-trial and follow-up, 4 and 2 nonexercise cows were scored as moderately lame, respectively, whereas no exercise cows were scored as moderately lame. Moreover, enhanced tracking up, reduced asymmetry, and balanced weight bearing in the exercise group indicate not only a progression toward smoother, more fluid movement but also a restoration of walking confidence, highlighting the potential of exercise to elevate both mobility and welfare. Our research studied the impact of relatively short periods of daily outdoor access (1 h/d, 5 d/wk) on the gait of dairy cows, distinguishing it from most previous studies that focused on seasonal or year-round full-time outdoor access (Hernandez-Mendo et al., 2007; Olmos et al., 2009; Chapinal et al., 2013). While these studies found an association between outdoor access and improved hoof or leg conditions, these improvements were thought to be intrinsically linked to long-term exposure to outdoor conditions, such as more comfortable footing and softer surfaces providing better traction than most indoor housing features (Shepley and Vasseur, 2021b). These factors may have influenced the significance of our results, as all cows, regardless of treatment group, still spent the majority of their time indoors.

**Figure 2:**
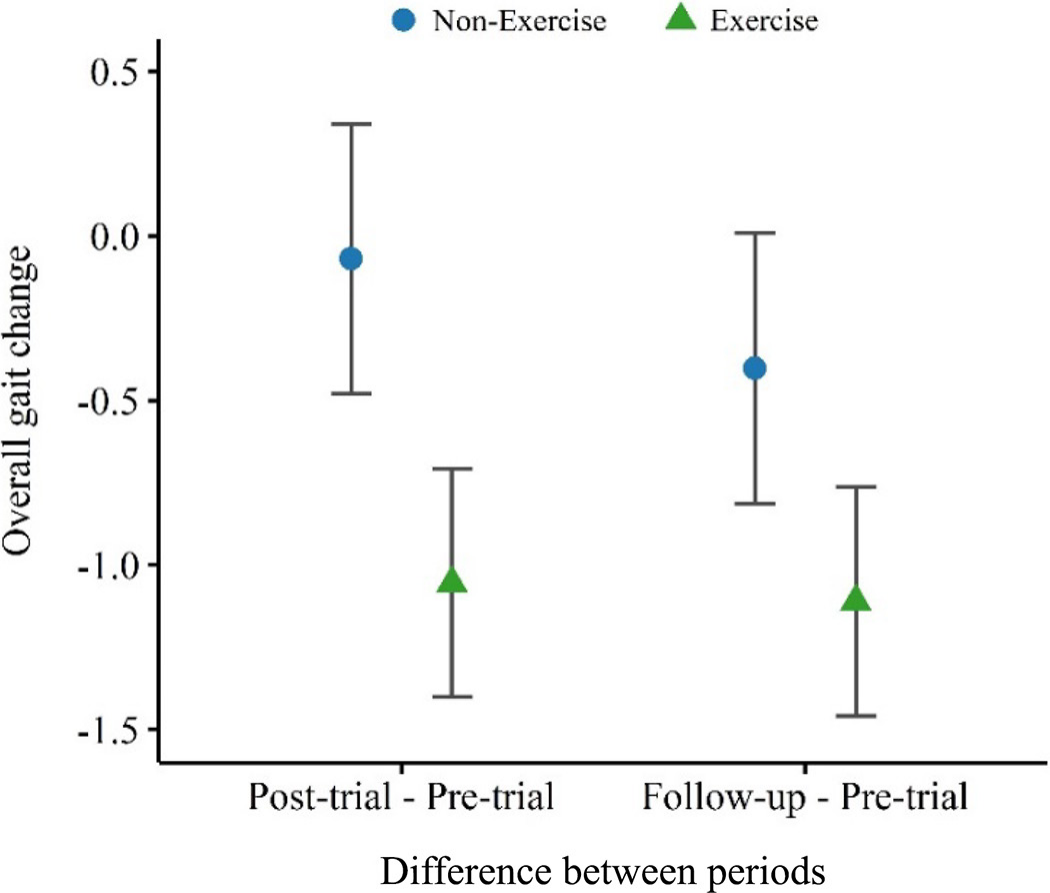
Changes in overall gait score between periods (post-trial – pre-trial and follow-up – pre-trial) for both treatment groups, nonexercise and exercise, illustrated with LSM (points) and SE bars.

**Figure 3:**
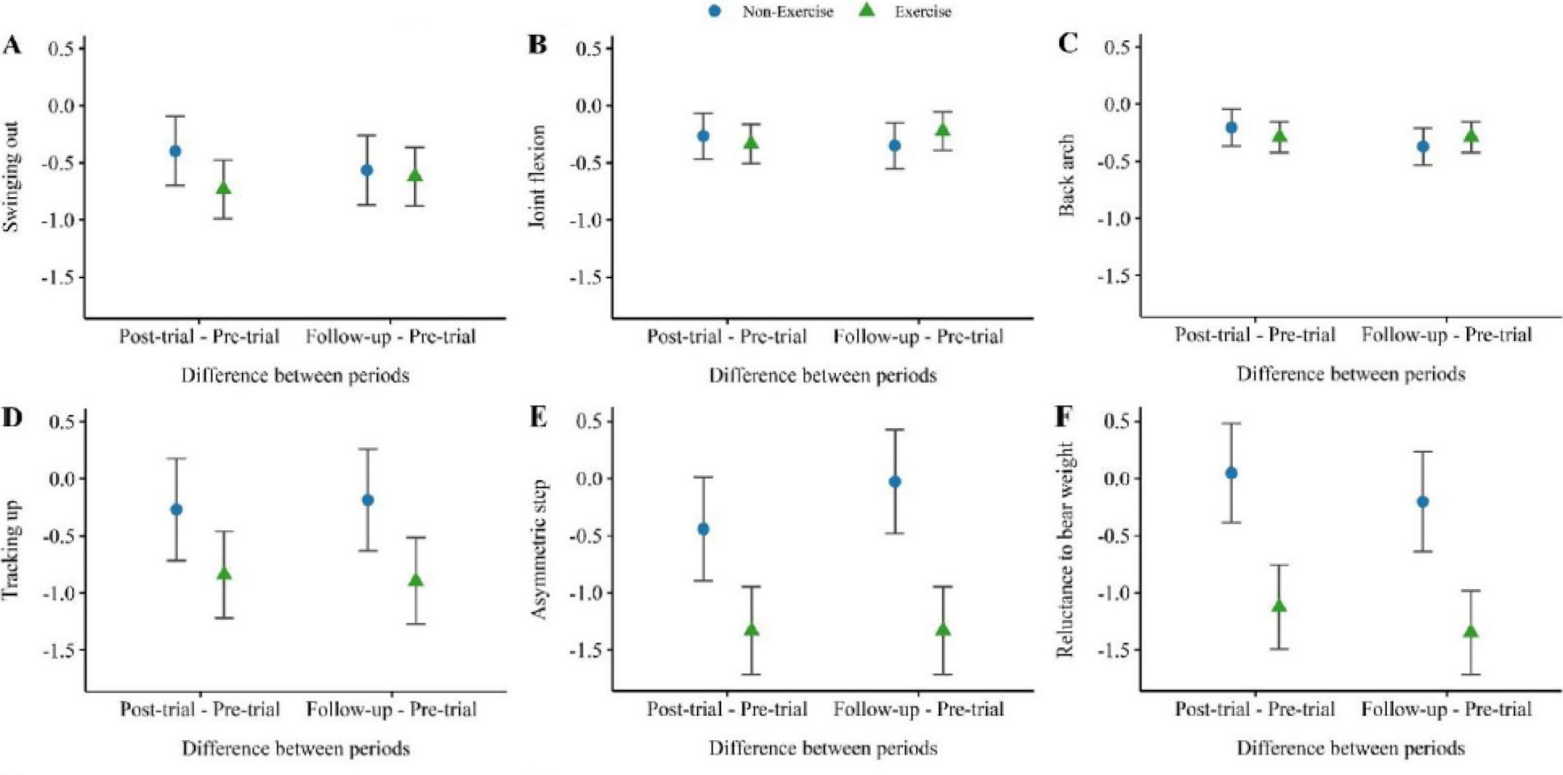
Changes in scores for 6 gait attributes: (A) swinging out, (B) joint flexion, (C) back arch, (D) tracking up, (E) asymmetric step, and (F) reluctance to bear weight, between periods (post-trial to pre-trial and follow-up to pre-trial). The data are depicted for both treatment groups, nonexercise and exercise, using LSM (points) and SE bars.

Cows in our study had relatively low starting gait scores (i.e., sounder gait), limiting room for improvement compared with noticeably lame cows under the same conditions, resulting in small overall score changes. Traditional tools such as visual gait scoring, designed for routine farm management to identify lame versus nonlame cows, may not detect slight changes in mobility. Previous studies reporting significant results included larger samples of both lame and nonlame cows (Hernandez-Mendo et al., 2007; Olmos et al., 2009; Chapinal et al., 2013). In contrast, our study had a smaller sample size and excluded obviously lame cows (score of 4 or higher) during the selection process, influencing the obtained results. The combination of limited room for gait improvement and a smaller sample size likely hindered our ability to identify statistically significant differences, despite noticeable improvements in exercise cows (a 1-point numerical score improvement after 5 wk of exercise exposure, maintained 8 wk post-trial). Our results align with Shepley and Vasseur (2021a), which also found numerical gait improvements over time, with only joint flexion showing a significant difference between treatments for cows with alternative housing during their dry period, whereas cows restricted to their stalls worsened over time for those same attributes. Future studies using advanced technological tools designed for gait analysis, offering greater sensitivity and objectivity, could provide a more comprehensive understanding of the observed changes beyond what human observers can discern (Bradtmueller et al., 2023; Nejati et al., 2023).Clinical hoof assessment showed an overall lesion prevalence of 7.9% at the claw level, considering 8 claws per cow, during the 2 data collection periods. All observed lesions were sole hemorrhages (**SH**), located in zones 4 (37 claws) and 5 (1 claw). Of the 38 SH, half were on hind claws (17 lateral, 2 medial) and half on front claws (all medial). The distribution of lesion severity was as follows: 55.3% for score 1, 34.2% for score 2, 10.5% for score 3, and none for score 4. No significant changes in the prevalence of SH were observed for both exercise (7.50% to 6.67%; *P* = 0.58) and nonexercise cows (10% to 8.04%; *P* = 0.16) from pre-trial to follow-up. Furthermore, the severity of lesions did not show any significant differences across the periods or treatment groups (*P* > 0.05).

There is limited literature available reporting the claw-level prevalence of lesions in tiestall-housed cows, making it challenging to directly compare our results. An epidemiological study by Cramer et al. (2008) reported results from tiestall dairies, with a prevalence of 25.7% for any hoof lesions and 7.1% for SH at the cow level. When compared with other hoof lesions typically reported, SH is considered a less severe lesion (Nocek, 1997), and its mild scores are often underreported by hoof trimmers (Solano et al., 2016). Therefore, it is possible that our methodology, specifically designed to detect even mild SH with slight discolorations at the claw-level, resulted in a higher cow-level prevalence of SH (40%) in our study compared with the aforementioned epidemiological study.

The absence of significant effects of outdoor access on hoof lesions aligns with our hypothesis, which emphasizes the lack of a detrimental effect of partial outdoor access on hoof health. This finding supports the results of Loberg et al. (2004), who observed no significant impact of various levels of outdoor access on claw lesions in tiestall-housed cows. Bielfeldt et al. (2005) similarly found no significant differences in claw sole disorders between cows housed in tiestall barns with and without outdoor access for exercise. However, a literature review on the effect of movement opportunity on cow health and comfort showed that pasture access positively affects hoof health (Shepley and Vasseur, 2021b), possibly due to more comfortable footing on pasture and increased blood flow in the legs improving hoof health. The lack of significant positive changes in our study may be attributed to the fact that the cows began the trial with a low prevalence and severity of claw lesions, thus providing limited scope for improvement.

Using the direct thermal analysis method on the thermography images, we found no statistically significant differences between treatment groups and time (pre-trial vs. post-trial) for all thermal variables. All thermal variables were significantly affected by ambient temperature (*P* < 0.001), which is consistent with previous studies (Landgraf et al., 2014; Alsaaod et al., 2015). However, when using the normalization method, the analysis of normalized images revealed a significant interaction in kurtosis between treatment groups and weeks (*P* < 0.0001). Kurtosis represents the width of the tails, or tailedness, of a normal distribution curve. A higher kurtosis often appears as a sharper peak and longer tails, indicating a population with more extreme outliers, while a lower kurtosis often appears as a flatter curve with shorter tails and indicates a population with less extreme outliers, ultimately representing a more homogeneous population (Westfall, 2014). In our study, kurtosis values decreased for both nonexercise and exercise groups from pre-trial to post-trial. Within each week, treatment groups were only significantly different on pre-trial, such that exercise cows started the trial with a higher kurtosis value than nonexercise cows at pre-trial. Although the reduction in kurtosis between pre- and post-trial was higher in exercise (from 2.61 ± 0.04 to 2.44 ± 0.04) than in nonexercise cows (from 2.44 ± 0.04 to 2.35 ± 0.04), there are uncertainties regarding the effect of exercise on the kurtosis values. Indeed, kurtosis as sole metric may not be sufficient to interpret the results further.

One of the possible risk factors of providing outdoor access on cow's hoof health is the impact of walking on hard, rough, frozen, and uneven surfaces that could lead to mechanically induced laminitis (Shearer et al., 2015), which can be identified by hoof surface thermography even at early stages (Nikkhah et al., 2005; Wood et al., 2015). We hypothesized that outdoor access would not compromise cow's hoof health by causing laminitis-associated inflammation that would increase hoof surface temperature. Consistent with our hypothesis, the thermography results using both analytical approaches suggest that hoof surface temperature is not influenced by application of 1 h/d outdoor access.

Overall, this study examined if 5 wk of daily outdoor exercise affected gait and hoof health in tiestall-housed dairy cows. Both overall gait score and 3 key attributes improved after 5 wk and persisted 8 wk post-trial, though these changes were not statistically significant. The small sample size and low starting gait scores may have limited significant findings. As the study did not focus on outdoor access as an aid for lameness recovery, only cows with sounder gait were enrolled, leaving less room for improvement. Assessing gait alterations in cows with minor gait issues requires more precise and objective methods than visual gait scoring, as subtle changes can often be overlooked by visual observation. Daily outdoor exercise had little impact on hoof health. No significant changes in hoof lesions or thermography, intended to detect laminitis-related temperature increases, were found. Future research should delve deeper into thermography and use advanced technology like kinematics to detect possible detailed and subtle enhancements in gait and hoof health due to increased mobility.
